# New ultrasonographic risk assessment of uterine scar dehiscence in pregnancy after cesarean section

**DOI:** 10.1007/s10396-022-01265-9

**Published:** 2022-12-19

**Authors:** Kosuke Kawakami, Toshiyuki Yoshizato, Yusuke Kurokawa, Naofumi Okura, Kimio Ushijima

**Affiliations:** 1 Department of Obstetrics and Gynecology, National Hospital Organization Kokura Medical Center, Kitakyushu, Japan; 2 Department of Obstetrics and Gynecology, School of Medicine, Kurume University, 67 Asahi-machi, Kurume, 830-0011 Japan

**Keywords:** Cesarean section, Dehiscence, Risk assessment, Transvaginal ultrasonography, Uterus

## Abstract

**Purpose:**

We performed a new ultrasonographic risk assessment of uterine scar dehiscence, which is a potential risk factor for uterine rupture, in pregnancy after cesarean section. We attempted to shed light on the natural course of the change in the lower uterine segment by means of a longitudinal investigation through quantitative and qualitative evaluations.

**Methods:**

This retrospective single-center study involved 31 women with a normal singleton pregnancy delivered by elective cesarean section between 2020 and 2021, with all women showing a “niche” in the lower uterine segments. The lower uterine segments were assessed qualitatively and quantitatively using transvaginal ultrasonography at 16–21, 22–27, and 28–33 weeks of gestation, and subjects were divided into two groups: those with uterine dehiscence (12 women) and those without uterine dehiscence (19 women), depending on the gross findings of the lower uterine segments at cesarean section. Analyses were performed using Wilcoxon’s rank-sum and Mann–Whitney *U* test with a significance level of *P* < 0.05.

**Results:**

The lower uterine segments changed from V-shaped to U-shaped to thin as gestation progressed and was more prominent in the uterine dehiscence group, occurring mostly at 22–27 weeks. At 22–27 weeks, the median myometrial thickness in the uterine dehiscence group was lower than in the group without uterine dehiscence (*P* = 0.0030). Thinning of the lower uterine segments had moved the cephalad at 22–27 and 28–33 weeks in cases with and without uterine dehiscence.

**Conclusion:**

A model of morphological changes in the niche was constructed based on qualitative and quantitative assessments. The morphological changes and actual thinning of the lower uterine segments were prominent in the second trimester in women considered to have uterine scar dehiscence.

## Introduction

Since “once a cesarean, always a cesarean” was advocated by Cragin [1] approximately 100 years ago, the delivery management of pregnancies after cesarean section (CS) has not been resolved. In 1988, the American College of Obstetricians and Gynecologists recommended vaginal birth and advocated a trial of labor after cesarean delivery (TOLAC) in an attempt to reduce the steady rise in CS rates [2]. However, TOLAC is associated with an increased incidence of uterine rupture and neonatal ischemic brain injuries [3]. The rates of CS have risen in recent decades [4]. Consequently, various complications related to repeated CS deliveries, such as placenta accreta spectrum disorders and cesarean scar syndrome, have been observed [5].

Examination of patients for potential risk factors is vital to reduce adverse outcomes from TOLAC. Many investigators have examined risk factors for uterine rupture using the clinical background of women undergoing TOLAC [6] and the prediction of uterine rupture through observation of the lower uterine segment (LUS) by ultrasonography during pregnancy.

Imaging studies have focused mostly on the thickness of the LUS during the third trimester [7–9]. However, there is still no satisfactory method of predicting uterine rupture in patients with TOLAC. The positive predictive values remain low, and the negative predictive values are not high enough for decision-making [9]. One of the reasons for the low positive predictive values is thought to be an overestimation of a thin LUS because such regions are physiologically extended and compressed by parts of the descended fetus during the third trimester. In addition, quantitative evaluation of a thin LUS is not always reliable because the measurement values are often beyond the resolution of ultrasonography. To improve the prediction rates for uterine rupture in patients undergoing TOLAC, the natural course of myometrial thinning of the LUS before the third trimester should be clarified.

In this study, we conducted a longitudinal investigation through quantitative and qualitative evaluations of the LUS in pregnancy after CS, from the second trimester to the first half of the third trimester.

## Materials and methods

### Subjects

Among a total of 114 deliveries at the Kokura Medical Center between December 2020 and March 2021, CS was performed in 37 women because of previous cesarean deliveries. This study involved 31 women with a singleton pregnancy with no maternal or fetal complications and in whom a niche in the LUS was found on transvaginal ultrasonography performed during the first trimester [10]; all women underwent elective CS at ≥ 37 weeks of gestation. In all cases, a lower uterine transverse incision was performed at the previous CS and repaired with double-layer absorbable sutures. No women had a history of uterine rupture.

The gestational age was estimated based on the crown–rump length at 9–11 weeks of gestation. The LUS was evaluated once at 16–21, 22–27, and 28–33 weeks of gestation by one examiner (K.K.). In all women, the placenta was not located on the anterior LUS, and the cervical length was > 25 mm at the time of observation.

### Data acquisition and parameter definitions

Prior to the examinations, the women were asked to wait until they felt the need to urinate. Ultrasound observations of the sagittal section of the anterior LUS were made with a transvaginal probe equipped with ultrasonography (Voluson P8; GE Healthcare, Tokyo, Japan). Measurements were made when the estimated bladder volume was 100–200 mL according to Haylen’s formula [11]. Images were magnified so that each LUS was visualized from the lowest point of the bladder to approximately 5 cm from that point. When localized uterine contractions were observed, image acquisition was delayed until the uterine contractions ceased.

**Fig. 1:**
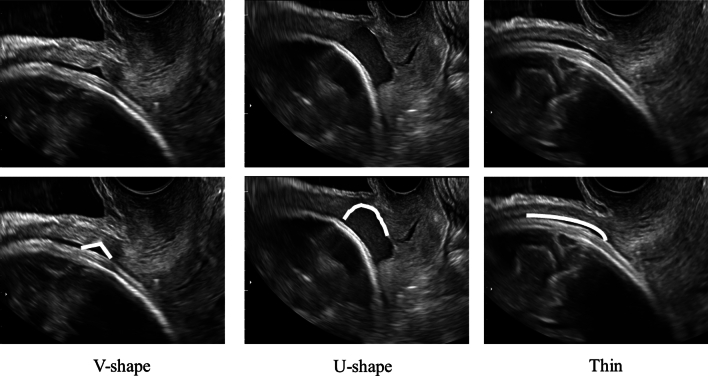
Classification of ultrasonographic images of the lower uterine segment

**Fig. 2:**
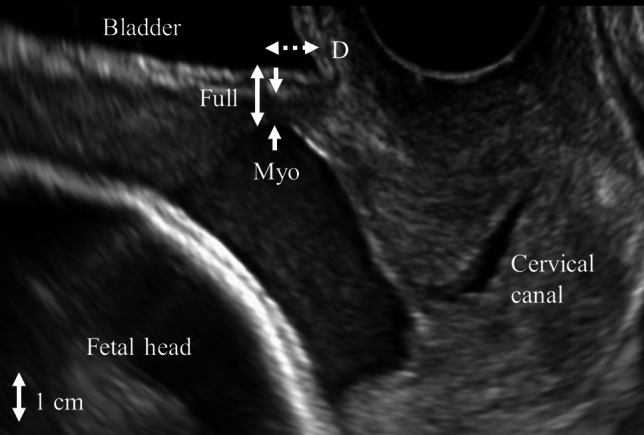
The definitions of myometrial thickness (Myo), full lower uterine segment (Full), and the distance from the lowest point of the bladder to the thinnest point of the lower uterine segment (D) are indicated by arrows

Analyses of ultrasonographic images were made offline by another examiner (T.Y.). For qualitative analysis, the morphology of the LUS was classified into three forms: V-shaped, U-shaped, and thin (Fig. 1) [12]. For quantitative analysis, the thinnest part of each LUS was measured by the myometrial thickness (only myometrium) and full LUS (all layers from the myometrium to the bladder wall), and the location of the thinnest part of each LUS was measured as the distance from the lowest point of the bladder (Fig. 2).

In this study, the gross findings of the LUS were divided into four classes based on the criteria described by Qureshi et al. [13]. The gross findings of the LUS at the time of CS were classified as class I: normal myometrium, class II: thinning of the myometrium but no fetal head (or other parts) visible, class III: thinning of the myometrium with fetal head (or other parts) visible, and class IV: uterine rupture. All evaluations to determine the classification were performed by qualified senior obstetricians. Classes I, II, and III were present in 16, 3, and 12 women, respectively. No women were characterized as class IV. Women evaluated as class III (12 cases) were considered to have uterine dehiscence, and those evaluated as class I and II (19 cases) were considered to be without uterine dehiscence.

### Analytical methods

First, the natural course of the morphological changes of the LUS in all cases of advanced gestation was examined by qualitative and quantitative analyses. Second, the natural course of the morphological changes of the LUS in all cases of advanced gestation with and without uterine dehiscence was examined by qualitative and quantitative analyses. Third, inter-group comparisons of ultrasound measurements of the LUS during the individual study periods were made between cases with and without uterine dehiscence.

### Statistical analyses

Intra- and inter-group comparisons of the quantitative analyses were performed using Wilcoxon’s rank-sum test and the Mann–Whitney *U* test, respectively, with a significance level of *P* < 0.05.

Before conducting this study, we calculated the sample size necessary for analysis. According to a report by Qureshi et al. regarding the incidence of uterine scar dehiscence and rupture in pregnancy after CS, 67.8%, 28.6%, and 3.6% of women were without uterine dehiscence, with uterine dehiscence, and with uterine rupture, respectively, and the ratio of women with uterine dehiscence or uterine rupture to women without uterine dehiscence was 0.47 [13]. With an α error of 0.05 and (1-β) error of 0.8, the sample size was calculated to be nine women without uterine dehiscence and 19 women with uterine dehiscence; thus, the number of women in the present study was proven to be adequate. The aforementioned analyses were performed with EZR (Saitama Medical Center, Jichi Medical University, Saitama, Japan) and a graphical user interface for R (www.r-project.org).

### Ethical considerations

This study was approved by the Institutional Review Board of the Kokura Medical Center (REC #2020–018; 19 November 2020). All procedures performed in this study involving human participants followed the ethical standards of the institutional and national research committees and the 1964 Helsinki declaration and its subsequent amendments or equivalent ethical standards. Informed consent was not required because of the retrospective study design.

## Results

### Clinical backgrounds of the women

The clinical backgrounds of the women are shown in Table 1. The only parameter that showed a significant difference between the groups was the inter-delivery interval between the most recent CS and the index pregnancy examined in the current study (*P* = 0.0074).

**Table 1:** Clinical backgrounds of the women in this study

		All cases (*n* = 31)		With uterine dehiscence *(n* = *12)*		Without uterine dehiscence *(n* = *19)*		*P *value
Maternal age^a^	(years)	34	(32–38)	33	(32–36)	35	(32–39)	*0.43*
Parity^a^	(*n*)	1	(1–2)	2	(1–2)	1	(1–2)	*0.23*
Gestational age at CS^a^	(weeks)	37	(37–38)	37	(37–37)	37	(37–38)	*0.23*
Body mass index^a^	(kg/m^2^)	21	(19.1–23.8)	20	(18.7–22.0)	21.4	(20.1–26.0)	*0.12*
Smoking habit^a^	(*n*)	0	(0–0)	0	(0–0)	0	(0–0)	−
Birth weight^a^	(g)	2643	(2441–2960)	2,593	(2404–2960)	2,697	(2441–2951)	*0.65*
Number of previous CS								
1	(*n*)	24	77.4%	9	75.0%	15	78.9%	*0.8*
> 2	(*n*)	7	22.6%	3	25.0%	4	21.1%	
Number of previous TVD								
0	(*n*)	25	80.6%	8	66.7%	17	89.5%	*0.17*
> 1	(*n*)	6	19.3%	4	33.3%	2	10.5%	
Inter-delivery interval from the most recent CS^a^	(days)	801	(527–1386)	603	(359–798)	1281	(618–1667)	***0.0074***
Gestational age at previous CS^a^	(weeks)	37	(36–38)	37	(36–37)	38	(37–38)	*0.18*
Emergent CS in the most recent delivery	(*n*)	14	45.2%	5	41.7%	9	47.4%	> *0.99*

Bold italics mean a significance of *P*<0.05

*CS*, cesarean section; *TVD*, transvaginal delivery

^a^Median (range)

### Qualitative and quantitative evaluations of longitudinal changes in the lower uterine segment in all cases

**Fig. 3:**
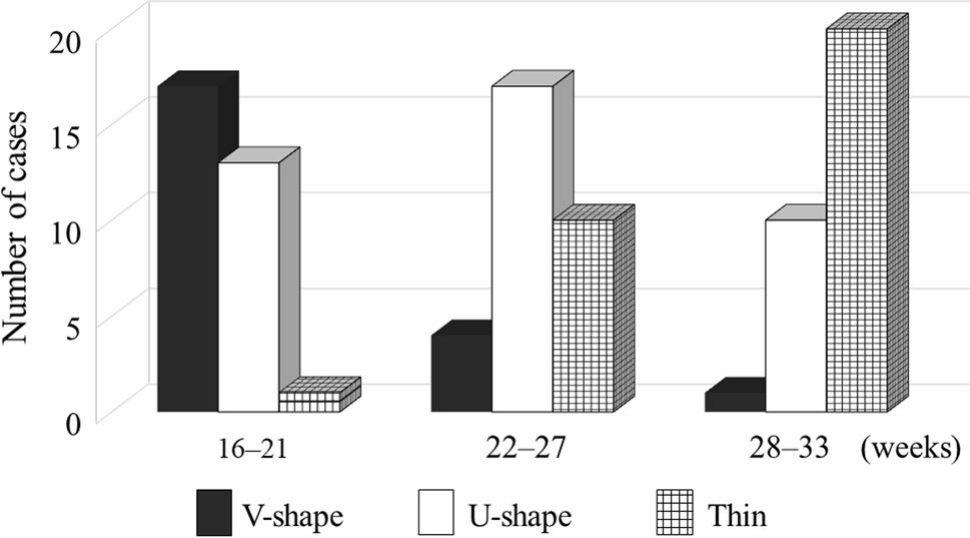
Changes in the number of women showing three different morphological patterns in the lower uterine segment in all cases

Longitudinal observations of qualitative assessments showed that the morphology of the LUS changed from V-shaped to U-shaped to thin as the gestational age progressed (Fig. 3).

**Fig. 4:**
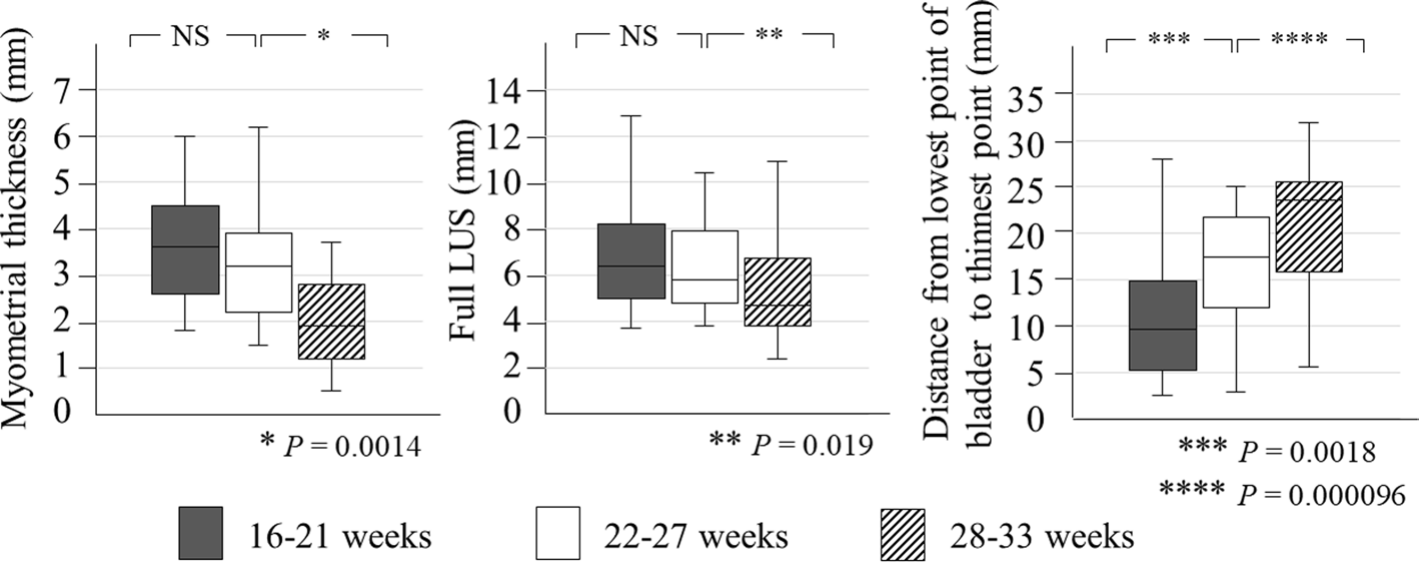
Quantitative evaluations of the longitudinal change in the lower uterine segment in all cases. Bars indicate the median and lower and upper quartiles. NS, not significant

The quantitative assessment showed that the LUS thickness became significantly thinner from gestational weeks 22–27 to 28–33 for the myometrial thickness and full LUS (*P* = 0.0014 and 0.019, respectively), and the location of LUS thinning moved the cephalad from the lower end of the bladder from gestational weeks 16–21 to 22–27 and from gestational weeks 22–27 to 28–33, with significant differences between the two study periods (*P* = 0.0018 and 0.000096, respectively) (Fig. 4).

### Qualitative and quantitative evaluations of longitudinal changes in the lower uterine segment in cases with and without uterine dehiscence

**Fig. 5:**
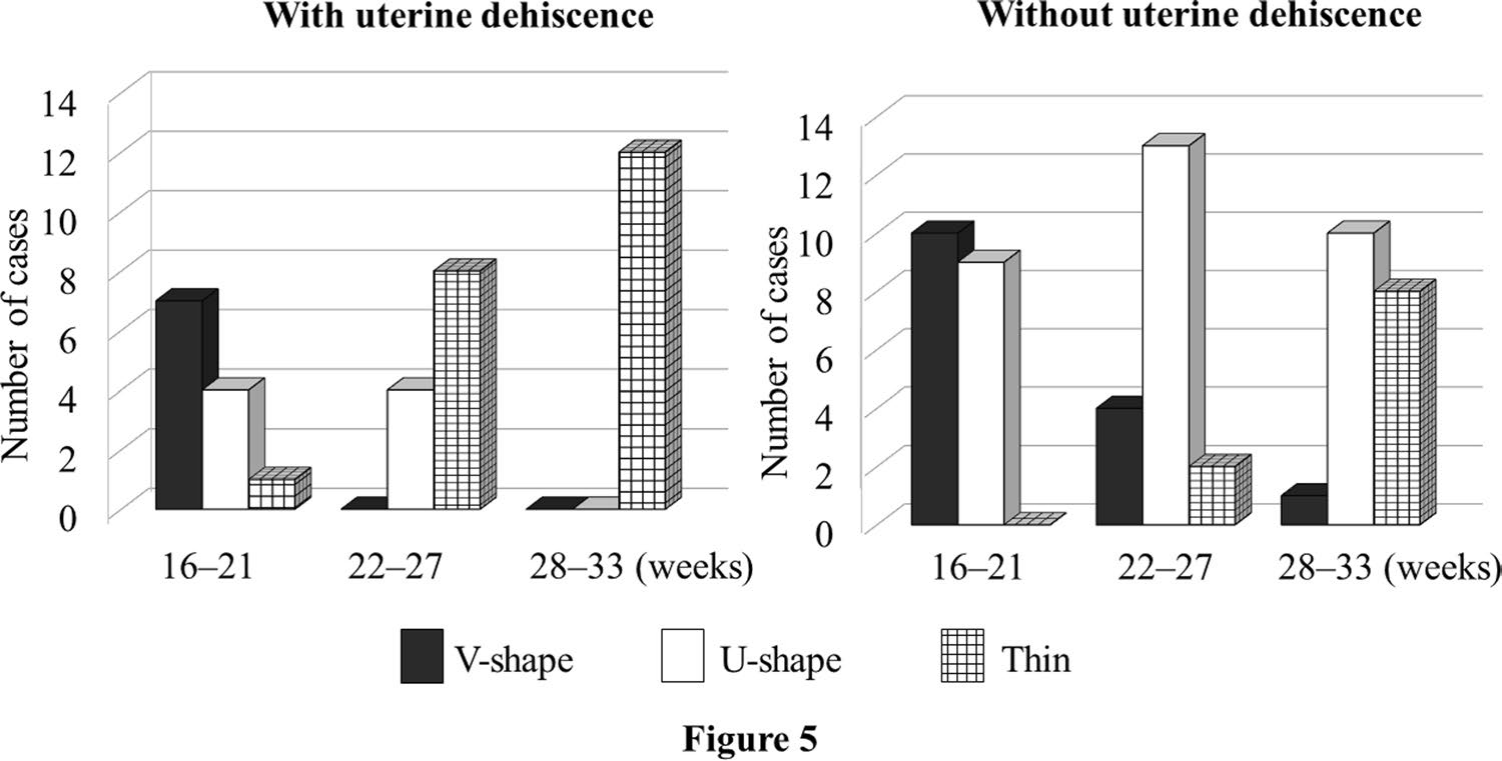
Changes in the number of women with three different morphological patterns in the lower uterine segment in cases with or without uterine dehiscence

In cases with uterine dehiscence, 66.7% (8/12) and 100.0% (12/12) of LUSs became thin by 27 and 33 weeks of gestation, respectively (Table 2 and Fig. 5). In cases without uterine dehiscence, 10.5% (2/19) and 42.1% (8/19) of LUSs became thin by 27 and 33 weeks of gestation, respectively.

**Table 2:** Changes in patterns of ultrasonographic images of the lower uterine segment in cases with and without uterine dehiscence

Morphological patterns of the LUS			With uterine dehiscence	Without uterine dehiscence	Total
16–21 weeks	22–27 weeks	28–33 weeks	(*n* = 12)	(*n* = 19)	
V-shape	V-shape	V-shape	0	1	1
V-shape	V-shape	U-shape	0	3	3
V-shape	U-shape	U-shape	0	4	4
U-shape	U-shape	U-shape	0	3	3
V-shape	V-shape	Thin	0	0	0
V-shape	U-shape	Thin	3	2	5
U-shape	U-shape	Thin	1	4	5
V-shape	Thin	Thin	4	0	4
U-shape	Thin	Thin	3	2	5
Thin	Thin	Thin	1	0	1

*LUS*, lower uterine segment

Numbers indicate the number of women

**Fig. 6:**
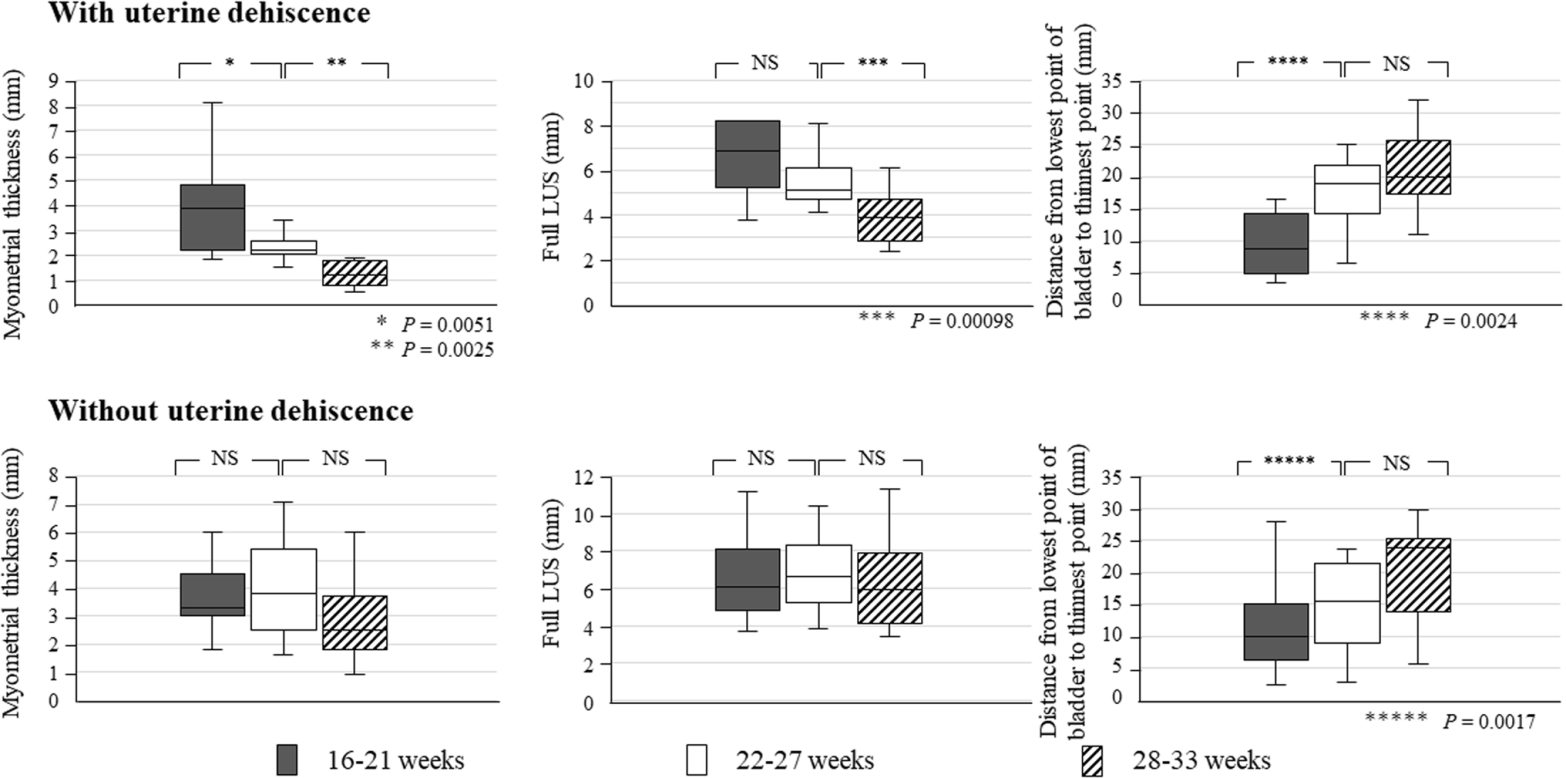
Quantitative evaluations of cases with or without uterine dehiscence. Changes in myometrial thickness, full LUS, and the distance from the lowest point of the bladder to the thinnest point of the lower uterine segment. Bars indicate the median and lower and upper quartiles. NS, not significant

In cases with uterine dehiscence, the myometrial thickness had thinned from 16–21 (median, 3.9 mm; range, 2.2–4.4 mm) to 22–27 weeks (median, 2.2 mm; range, 2.1–2.5 mm) (*P* = 0.0051) and from 22–27 to 28–33 weeks (median, 1.2 mm; range, 1.0–1.7 mm) (*P* = 0.0025) (Fig. 6). In contrast, in cases without uterine dehiscence, the myometrial thickness showed no differences from 16–21 to 22–27 weeks and from 22–27 to 28–33 weeks. In cases with uterine dehiscence, the full LUS did not thin from 16–21 weeks to 22–27 weeks (*P* = 0.091) but showed significant thinning from 22–27 weeks (median, 5.1 mm; range, 4.8–6.0 mm) to 28–33 weeks (median, 3.9 mm; range, 2.9–4.5 mm) (*P* = 0.00098). In contrast, in cases without uterine dehiscence, there were no differences in the full LUS from 16–21 weeks to 22–27 weeks and from 22–27 weeks to 28–33 weeks. In cases with uterine dehiscence, the location of LUS thinning moved away from the lower end of the bladder from 16–21 (median, 8.8 mm; range, 4.8–14.3 mm) to 22–27 weeks (median, 18.9 mm; range, 14.9–21.7 mm) (*P* = 0.0024) but did not change thereafter. In contrast, in cases without uterine dehiscence, the location of LUS thinning did not change between 16–21 and 22–27 weeks but thereafter shifted away from the lower end of the bladder until 28–33 weeks (22–27 weeks: median, 15.5 mm; range, 10.5–20.7 mm and 28–33 weeks: median, 23.8 mm; range, 14.3–24.9 mm) (*P* = 0.0017).

### Inter-group comparison of ultrasound measurements of the lower uterine segment between cases with and without uterine dehiscence

At 16–21 weeks of gestation, there were no differences in the myometrial thickness or full LUS between cases with and without uterine dehiscence (Table 3). At 22–27 weeks of gestation, the median myometrial thickness in cases with uterine dehiscence (2.2 mm; range 2.1–2.5 mm) was lower than that in cases without uterine dehiscence (3.8 mm; range 2.9–4.9 mm) (*P* = 0.0030). However, there was no difference in the full LUS. At 28–33 weeks of gestation, the myometrial thickness and full LUS in cases with uterine dehiscence were lower than those in cases without uterine dehiscence (*P* = 0.0011 and 0.0037, respectively).

**Table 3:** Inter-group comparisons of ultrasonographic measurements of the lower uterine segment between cases with and without uterine dehiscence

Gestation (weeks)	Thickness (mm)	With uterine dehiscence (*n* = 12)		Without uterine dehiscence (*n* = 19)		
		Median	Range	Median	Range	*P* value
16–21	Myometrial	3.9	2.2, 4.4	3.3	3.0, 4.3	*0.97*
	Full LUS	6.9	5.3, 8.2	6.1	4.9, 7.9	*0.63*
22–27	Myometrial	2.2	2.1, 2.5	3.8	2.9, 4.9	***0.003***
	Full LUS	5.1	4.8, 6.0	6.6	5.2, 8.3	*0.064*
28–33	Myometrial	1.2	1.0, 1.7	2.5	1.9, 3.6	***0.0011***
	Full LUS	3.9	2.9, 4.5	5.9	4.1, 7.7	***0.0037***

Bold italics mean a significance of *P*<0.05

*LUS*, lower uterine segment

## Discussion

The presence of a “niche,” namely the formation of a wedge in the myometrium in the anterior LUS after CS, is considered a risk factor for uterine rupture in subsequent pregnancies [14, 15]. Therefore, the women in the present study were limited to those in whom niche formation was recognized in the first trimester and in whom longitudinal observations of the LUS were made from the second to third trimesters using transvaginal ultrasonography.

The morphological changes of the LUS during the second and third trimesters can be influenced by various factors, including localized myometrial contractions; compression by adjacent organs, such as the bladder and fetal parts; and the physiological elongation of the LUS with advancing gestation [16]. Therefore, to minimize such effects, we observed LUS measurements in the absence of localized uterine contractions and with the bladder capacity kept constant for each case. In addition, we excluded observation periods after 34 weeks of gestation to avoid the confounding effects of descending fetal body parts.

**Fig. 7:**
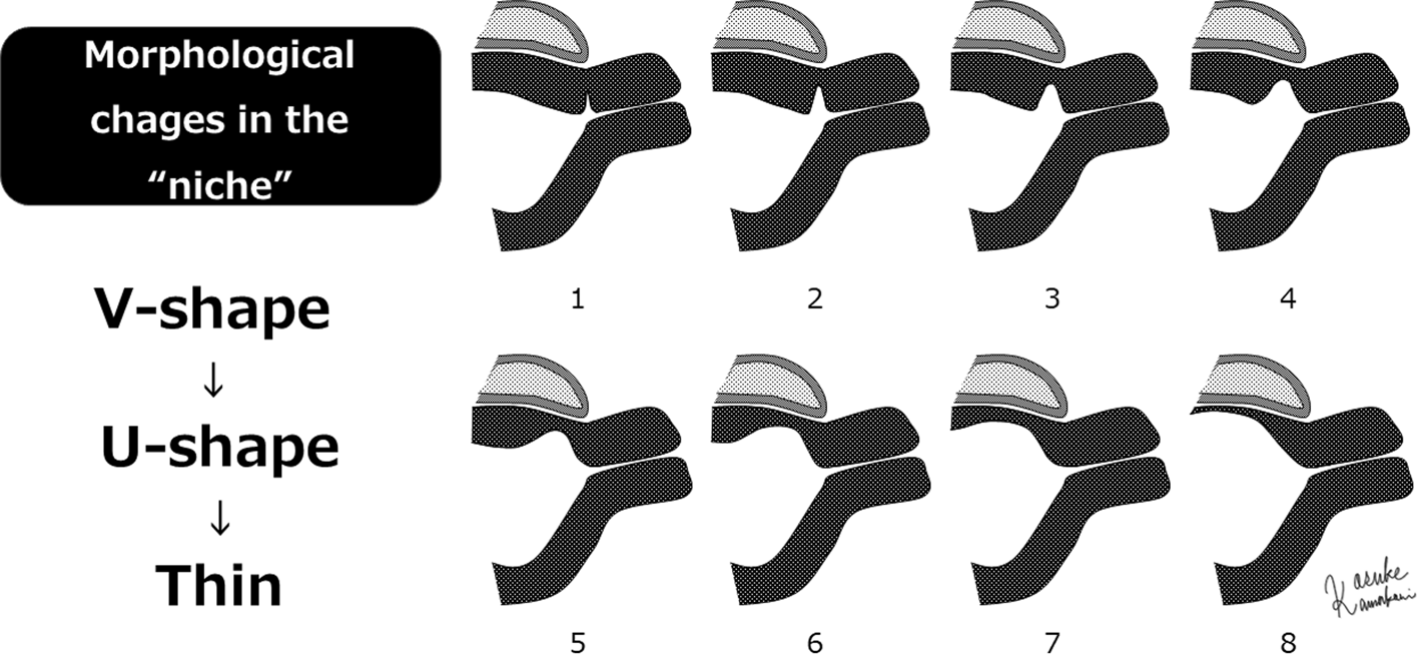
Proposed model of the natural course of morphological changes in the niche with advanced gestation

In our study, we demonstrated that the niche morphology changed from V-shape to U-shape to thin over time by qualitatively and quantitatively assessing the niche changes before the third trimester. This quantitative analysis allowed us to develop a natural course model of niche changes. Based on the study findings, we propose a natural course in the morphological changes of the niche during pregnancy in which the niche observed in the first trimester develops from a V-shape to a U-shape to thin with advanced gestation during the second to third trimesters (Fig. 7).

In an additional study, we compared the clinical backgrounds between cases with and without uterine dehiscence and found a significant difference in the inter-delivery interval between the preceding CS and the index pregnancy between the groups. Although this study did not include any cases of uterine rupture, the test results were consistent with previous reports on the risk of uterine rupture [17, 18].

Although there have been many reports of observation of the LUS in pregnancy after CS in cases near term, few studies have assessed LUS changes with advanced gestation before the third trimester. Naji et al. [19, 20] reported that women with progressive LUS thinning from the first to second trimesters were more likely to experience TOLAC failure. Gotoh et al. [21] reported that the thickness of the LUS in women who had previously undergone CS was thinner at the third trimester than in women without a history of CS; they also stated that LUS thinning in the high-risk group was measured more accurately during the second trimester than at term. Although our study did not include any cases with uterine rupture, this morphological change might occur at earlier gestational weeks in cases with uterine dehiscence. We speculated that the reason for the early morphological changes in cases with uterine dehiscence was less residual myometrium and more scar tissue, which may have decreased the elasticity of the tissues, making the morphological changes more pronounced before the physiological stretching of the LUS progressed.

Comparison between the myometrial thickness and full LUS as a method of measuring the LUS remains controversial. Most previous studies used transabdominal ultrasonography during the third trimester [9]. Direct measurements of myometrial thickness seem to be superior to full LUS; however, the reproducibility is less reliable because the LUS becomes thinner during the third trimester, especially near term, often making measured values beyond the resolution of ultrasonography. In our study, inter- and intra-group comparisons revealed no differences between the myometrial thickness and full LUS in the third trimester in terms of the prediction of actual LUS thinning during CS, but the myometrial thickness was superior for measurements during the second trimester. This suggests that the myometrial thickness is preferable when measurements are carried out in the second trimester and longitudinally from the second to third trimesters.

The strength of our current study is that the myometrial thickness and full LUS were measured longitudinally from the second trimester to the early third trimester using transvaginal ultrasonography, which has a higher resolution than transabdominal ultrasonography. These changes were incorporated into the LUS morphology, and we measured LUS thickness to create a model of the natural course of changes in the niche.

Additional analyses indicated that cases with uterine dehiscence might show morphological changes in the LUS earlier in gestation than those without uterine dehiscence. The model of the natural course of changes in the niche might provide a new method for assessing thinning of the lower uterus in pregnancy after CS.

The limitations of this study were its retrospective design and the inclusion of women with niche formation in the LUS and no cases of uterine rupture. However, we assume that our study will help establish criteria that will allow TOLAC to be performed safely. Large-scale prospective studies are necessary to assess the safety of TOLAC.

## Conclusions

We reported the natural course of morphological changes in the niche during pregnancy with advanced gestation. The morphological changes and actual thinning of the LUS were prominent in the second trimester in women considered to be at risk of uterine dehiscence. Measurement of the myometrial thickness is preferable to measurement of the full thickness for the quantitative evaluation of such women.
